# Supplementary material for: Serum sodium on admission affects postoperative in-hospital mortality in acute aortic dissection patients
Source: PLoS One. 2021 Dec 15;16(12):e0261168. doi: 10.1371/journal.pone.0261168 (PMC8673641; doi:10.1371/journal.pone.0261168)
Supplement: Attachment — Submitted filename: Response to Reviewers.docx [file pone.0261168.s002.docx]

Dear Michele Provenzano:

 Thank you very much for giving us an opportunity to revise our manuscript entitled" **Serum sodium on admission affects postoperative in-hospital mortality in acute aortic dissection patients**" (Manuscript No.: PONE-D-20-40304).

We have carefully read your letter and the reviewers’ comments and have performed a number of new experiments and revised the manuscript accordingly. All changes have been highlighted in red color in the revised manuscript. Point by point responses to the reviewers’ comments are listed below this letter.

Thank you and all the reviewers for the kind advice.

We hope that the revised version of the manuscript is acceptable for publication in PLoS One. Look forward to hearing from you.

Best wishes,

Yours sincerely,

Dong Ma

Point-by-point response to referees.

Reviewer #1:
Major concerns:

Q: (1) *In abstract, serum sodium has been splitted into quartiles according to distribution. Was Q1 considered the reference in che cox model ? please specify this information since it has been stated that hazard ratio in Q3 and Q4 was increased but it was not reported the reference category. Please remember that the hazard ratio is a measure of relative risk (in time-to-event analyses)*

A: Thanks for your careful review. According to your suggestion, we emphasized that Q1 is the reference in che cox model and marked in red word in revised manuscript.

*Q: (2) Methods: how variables have been added to the model 2? based on the plausibility of their association with short term mortality ? please clarify*

A: Thanks for your question. According to reference (Huaping He, Xiangping Chai, Yang Zhou, et al. Association of lactate dehydrogenase with in-hospital mortality in patients with acute aortic dissection: a retrospective observational study. Int J Hypertens. 2020, 2020:1347165.), we adjusted for age, gender, Stanford type, hypertension, diabetes, CAD, smoking, drinking, serum glucose, α-HBDH in model 2 to further analyse the association with short term mortality.

*Q: (3) usually the variable sodium has a biphasic association with risk (U-shaped). This means that risk increases for both low and high sodium values. Was the linearity of effect of sodium on?*

A: Thanks for your valuable suggestion. In this study, we only found that there was an increase of sodium levels in serum from AAD patients, and this increased value was not linear association with the endpoint tested (after surgery). Additionally, in the most studies, the increased sodium level is general in clinical reports, which is consistant with our result.

*Q: (4) Following the previous point, I would suggest Authors to do a sensitivity analysis by replacing the category Q1 with Q2 as reference for the Cox model*

A: Thanks for your suggestion. We performed strict inclusion and exclusion criteria to ensure the authenticity of the data. The methods used in the study are mature and reliable to ensure the accuracy of the results. Thus, we believe our conclusion is stable.

*Q: (5) I am not fully sure that Cox model is appropriate for such analysis with short-term follow-up (of some days). It seems that the event occurs almost in the same period of sodium measurement. Can Authors provide one or more references which may clarify this point?*

A: Thanks for your suggestion. There has been a reported paper on Cox model for short-term follow-up (Zhang S, Guo M, Duan L, et al. Development and validation of a risk factor-based system to predict short-term survival in adult hospitalized patients with COVID-19: a multicenter, retrospective, cohort study[J]. Crit Care, 2020, 24(1): 438.).

Reviewer #2:

*Q: (1). an English language editing*

A: Thank you for your valuable suggestion. We have changed the format as required. Please check.

*Q: (2). to review fig n. 2 (low quality image)*

A: Thank for your careful review. We uploaded a high quality image of fig2. Please check.

*Q: (3). to write the small cohort of patients in the study limitations*

A: Thanks for your suggestion. The first limitation was a single-center study, which is not enough to get strong warrant to prove serum sodium levels independently associated with in-hospital mortality of AAD patients. The other limitation is the short-term studies could not better assess the effect of serum sodium on in-hospital mortality in AAD patients. We have added these limitations in discussion.
