# Supplementary material for: Serum sodium on admission affects postoperative in-hospital mortality in acute aortic dissection patients
Source: PLoS One. 2021 Dec 15;16(12):e0261168. doi: 10.1371/journal.pone.0261168 (PMC8673641; doi:10.1371/journal.pone.0261168)
Supplement: Attachment — Submitted filename: Response to Reviewers2.docx [file pone.0261168.s003.docx]

Dear PLoS One Publications
 Thank you very much for giving us an opportunity to revise our manuscript entitled" **Serum sodium on admission affects postoperative in-hospital mortality in acute aortic dissection patients**" (Manuscript No.: PONE-D-20-40304).

We have carefully read your letter and the reviewer comments and have performed a number of new experiments and revised the manuscript accordingly. All changes have been highlighted in red color in the revised manuscript. Point by point responses to the reviewers’ comments are listed below this letter.

Thank you and all the reviewers for the kind advice.

We hope that the revised version of the manuscript is acceptable for publication in PLoS One. Look forward to hearing from you.

Yours sincerely,

Dong Ma

Point-by-point response to referees.

Reviewer #3:
Major concerns:

Q: (1) *One major concern is the proportional hazards assumption. There is huge difference between patients who survived and those who died (14 days vs 2 days). The authors should formally test the assumption before using the Cox model.*

A: Thanks for your careful review. In order to verify whether Cox proportional risk model would be available for the short-term survival analysis in AAD patients, proportional hazards assumption (PH) and schoenfeld residual method were performed to test the assumption. We plot the smooth curve residual diagram of Schoenfeld's residual with time, and the results showed no linear correlation between Schoenfeld's residual and time rank (Fig 4). Moreover, we tested that the correlation between Schoenfeld's residual and time rank, and found that Schoenfeld's residual was independent on time variables (Pearson correlation coefficient, r = -0.22, P = 0.864). Based on these two results of the smooth curve trend and the Pearson correlation from Schoenfeld's residual analysis, indicating that Cox model is available to the short-term survival analysis of patients with AAD.

Fig 4 Schoenfeld residual diagram

*Q: (2) Another concern is that this is an association study. There is no way to derive causality effect. Please remove any implication of causality.*

A: Thanks for your good suggestion. We modified these inappropriate words as required in revised manuscript. Please check.

*Q: (3). Please clarify how the variables were determined. For example, for the smoking, is it current smoker or past smoker? For drinking, how many drinks were used to determine the drinker status?*

A: Thanks for your careful review. In this study, smoking is defined as at least 1 cigarette daily for 1 year or more, and alcohol consumption is defined as at least once per week for a period of one month or longer. We added these in part of methods and marked in red word. Please check.

*Q: (4). The exclusion criteria included the serious lack of clinical data. How is it defined? What about other variables with a moderate missing rate? Were they excluded or imputed?*

A: Thanks for your suggestion. In this study, the severe lack of clinical data is defined as the absence of complete hospitalization records, involving lack of blood glucose, blood pressure and other important indicators associated with the known risk factors of AAD. Except for basic information and indicators associated with the known risk factors of AAD, the other defect data were considered to be a moderate missing for some individual patients, who were included in the study. We revised the exclusion criteria in the part of methods marked in red word.

*Q: (5) Did any patient die before the operation? If so, will it affect the association? What is the time interval between admission and the operation?*

A: Thanks for your suggestion. Aortic dissection is a very dangerous disease, especially Stanford A type. Once AAD patient is diagnosed, emergency surgical treatment should be firstly required in principle. Due to the deaths occurred quickly before or after admission, AAD patients’ serum sodium value and other relevant data could not be collected immediately, where 6 hours is the shortest operation interval after admission in our hospital, therefore, some patients who died before surgery were not involved in this study, and these defect data may affect the results of the study. This is a limit for the study added in discussion in red word.

*Q: (6). It seems that the variation of sodium levels is very small (SD=4.8). All four quartiles included some participants with normal sodium levels (Q1 : ≤ 136 mmol/L, Q2 : 137 – 138 mmol/L, Q3: 139 -140 mmol/L, Q4 : ≥141 mmol/L). What would be measurement variability?*

A: In this study, automatic electrolyte analyzer (Shenzhen Kangli AFT--500D) was used for the determination of serum sodium and other ions, with high accuracy and good precision. The CV value of Na detection was generally 0.60%, which is sufficient to meet the accuracy of this research. We marked this analyzer in the part of methods in red word.

*Q: (7). Can authors comment on the possible reasons why Q2 was not significant, but Q3 and Q4 were significant?*

A: Thank for your suggestion. It was possible that Q2 (137-138 mmol/L) slightly affected postoperative mortality in patients with AAD, but it was not independent related to postoperative death in patients with AAD (HR=1.718 95%CI 0.688-4.486, *P*=0.269).The results of Q3 and Q4 showed that when the serum sodium≥ 139mmoL/L, the postoperative in-hospital mortality of AAD patients may be increased significantly, there exists an important association between serum sodium and the postoperative in-hospital mortality in the study.

*Q: (8). In Figure 2, please indicate the number of events under the x-axis. Also it is unclear why there was a big drop of survival probability in Day 40 for Q4, especially given the median of 2 days in the non-survival group.*

A: Thanks for your suggestion. We revised image of fig2, and please check.

*Q: (9). In Figure 3, given that there is no direct connection between quarters, a barplot would be more appropriate.*

A: Thanks for your suggestion. We replaced figure 3 with a barplot.
